# BH3 mimetics suppress CXCL12 expression in human malignant peripheral nerve sheath tumor cells

**DOI:** 10.18632/oncotarget.14398

**Published:** 2016-12-31

**Authors:** Christopher D. Graham, Niroop Kaza, Hawley C. Pruitt, Lauren M. Gibson, Barbara J. Klocke, Lalita A. Shevde, Steven L. Carroll, Kevin A. Roth

**Affiliations:** ^1^ Department of Pathology, University of Alabama at Birmingham, Birmingham, Alabama, United States of America; ^2^ Medical University of South Carolina, Department of Pathology and Laboratory Medicine, Charleston, South Carolina, United States of America; ^3^ Columbia University Medical Center, Department of Pathology and Cell Biology, New York, United States of America

**Keywords:** MPNST, BH3 mimetic, CXCL12, PARP1, AT101

## Abstract

Malignant peripheral nerve sheath tumors (MPNSTs) are aggressive, Schwann cell-derived neoplasms of the peripheral nervous system that have recently been shown to possess an autocrine CXCL12/CXCR4 signaling loop that promotes tumor cell proliferation and survival. Importantly, the CXCL12/CXCR4 signaling axis is driven by availability of the CXCL12 ligand rather than CXCR4 receptor levels alone. Therefore, pharmacological reduction of CXCL12 expression could be a potential chemotherapeutic target for patients with MPNSTs or other pathologies wherein the CXCL12/CXCR4 signaling axis is active. AT101 is a well-established BCL-2 homology domain 3 (BH3) mimetic that we recently demonstrated functions as an iron chelator and thus acts as a hypoxia mimetic. In this study, we found that AT101 significantly reduces CXCL12 mRNA and secreted protein in established human MPNST cell lines *in vitro*. This effect was recapitulated by other BH3 mimetics [ABT-737 (ABT), obatoclax (OBX) and sabutoclax (SBX)] but not by desferrioxamine (DFO), an iron chelator and known hypoxia mimetic. These data suggest that CXCL12 reduction is a function of AT101's BH3 mimetic property rather than its iron chelation ability. Additionally, this study investigates a potential mechanism of BH3 mimetic-mediated CXCL12 suppression: liberation of a negative CXCL12 transcriptional regulator, poly (ADP-Ribose) polymerase I (PARP1) from its physical interaction with BCL-2. These data suggest that clinically available BH3 mimetics might prove therapeutically useful at least in part by virtue of their ability to suppress CXCL12 expression.

## INTRODUCTION

MPNSTs arise sporadically or in patients affected by neurofibromatosis type 1 (NF1) [1]. NF1 is the most commonly inherited cancer predisposition syndrome of the human nervous system, affecting 1 in 3500 newborns [2]. NF1 patients typically exhibit numerous benign neurofibromas, and 8 – 13% of patients will develop more aggressive MPNSTs [1]. Despite increased understanding of the underlying molecular mechanisms driving the development of MPNSTs, treatment advances have been limited. Surgery is the standard of care; however, complete tumor excision is often impossible as MPNSTs aggressively invade adjacent tissues and metastasize [3]. Further, radiation and chemotherapy are largely ineffective at promoting MPNST patient survival [4]. These factors result in a <50% five-year survival rate for MPNST patients [1] and MPNSTs remain the leading cause of death in patients with NF1 [5].

CXCL12 (stromal cell-derived factor-1, SDF-1) belongs to a class of small (8-12 kilodalton [kD]) cytokines, known as chemokines (chemotactic cytokines). Based on structure, CXCL12 is a member of the C-X-C sub-group of chemokines possessing a single, non-conserved amino acid residue (X) between the first N-terminal cysteine residues. Originally described as a homeostatic chemokine with a prominent role in the trafficking of hematopoietic cells, CXCL12 is now recognized to modulate multiple physiological processes via activation of its receptor, CXCR4 [5, 6]. Such pathways include activation of phospholipase C (PLC), regulation of adenylate cyclase and stimulation of kinase cascades such as phosphoinositide 3-kinase (PI3K), mitogen-activated protein kinase (MAPK) and c-Jun N-terminal kinase (JNK) [7, 8, 9, 10]. In addition to its physiological role, CXCL12/CXCR4 signaling has been implicated in driving tumor cell proliferation, survival and migration in various solid tumors [11, 12]. The original view of stromal cell-secreted CXCL12 acting on CXCR4-expressing tumor cells in a paracrine manner has evolved in recent years. Several studies have shown that some tumor cells, in addition to expressing CXCR4, can secrete CXCL12 and so activate an autocrine loop promoting growth and metastasis [6, 13]. Parada, *et al*'s discovery of this autocrine signaling axis’ involvement in NF1-derived MPNST cells provides an exciting opportunity to explore novel therapies targeting CXCL12/CXCR4 autocrine signaling to treat MPNST patients [6].

In addition to CXCR4, CXCL12 also binds a “decoy” receptor, CXCR7 [14]. While it is thought that CXCR7 does not induce the same cascade of signals mediated by CXCR4, CXCR7 has been shown to significantly increase cell proliferation and adhesion in various conditions [14, 15, 16, 17]. Speculation abounds as to the biological role(s) of CXCR7, from the idea that it sequesters CXCL12 to create a gradient leading to differential signaling through CXCR4 [18, 19], to the hypothesis that CXCR7 serves as a co-receptor for CXCR4 [20, 21]. Accordingly, inhibition of CXCR7 function may serve to increase the clinical efficacy of CXCR4 inhibitors such as AMD3100, as blockage of CXCR4 only partially inhibits tumor cell response to CXCL12 gradients in multiple animal models [22, 23, 24]. These observations strengthen the argument that inhibition of CXCL12 expression may complement or enhance the efficacy of clinically available CXCR4 antagonists such as AMD3100.

AT101 [(-)-gossypol acetic acid] is a modified enantiomer of the naturally occurring polyphenolic aldehyde gossypol, and has received significant attention as a potential chemotherapeutic agent in multiple tumor types [25]. The basis for AT101's anti-neoplastic activity has been largely attributed to its BH3 mimetic activity, which results in interaction and inhibition of anti-apoptotic B-Cell Lymphoma 2 (BCL-2) family proteins such as BCL-2, BCL-X_L_ and MCL-1 [25]. This BH3 mimetic property of AT101 has been shown to promote both apoptotic and non-apoptotic tumor cell death, *in vitro* and *in vivo* [26, 27]. We recently reported that AT101, independent of its BH3 mimetic property, acts as an iron chelator in established human MPNST cell lines [4].

In this report, we show that AT101 causes a significant reduction in CXCL12 mRNA and secreted protein in established human MPNST cell lines. This effect results from AT101's BH3 mimetic property rather than its iron chelation ability. Finally, we show that the BH3 mimetic ABT robustly increases PARP1 binding to the *CXCL12* promoter.

## RESULTS

### AT101 suppresses CXCL12 expression

**Figure 1:**
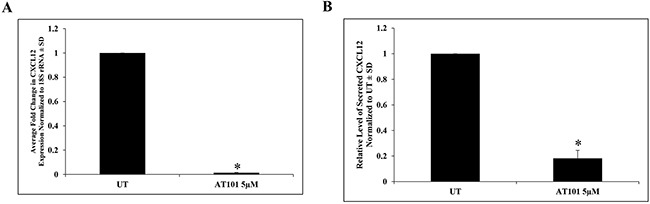
AT101 down-regulates CXCL12 in MPNST cells **A.** qRT-PCR analysis of AT101-treated T265-2c cells (5μM, 24h). **B.** AT101 treatment (5μM, 24h) resulted in a significant reduction of secreted CXCL12 protein in T265-2c cells as demonstrated by an ELISA. *p-value <0.05.

Because an active CXCL12/CXCR4 signaling pathway has been shown to mediate tumor cell proliferation, survival and migration in several tumor types including MPNSTs [6, 11, 12] and BH3 mimetics have been demonstrated to modulate CXCL12 transcription [28, 33], we sought to assess CXCL12 mRNA levels in T265-2c cells treated with AT101 (5μM for 24h) by quantitative real time PCR. We found that AT101 treatment resulted in a dramatic reduction of CXCL12 mRNA expression in T265-2c cells (Figure 1A, Supplementary Figure 4). CXCL12 is a chemotactic cytokine and is rapidly secreted, making it difficult to measure levels of intracellular CXCL12 in cell extracts. Accordingly, we performed an Enzyme-Linked ImmunoSorbent Assay (ELISA) on T265-2c culture media that had been treated with or without AT101 to assess whether treatment suppressed CXCL12 protein secretion as well as mRNA expression. Our data demonstrate that AT101 treatment (5μM for 24h) significantly decreased levels of secreted CXCL12 protein compared to untreated cells (Figure 1B Supplementary Figure 5). Our findings indicate that AT101 suppresses both CXCL12 expression and secretion in T265-2c MPNST cells. ABT, OBX, SBX and DFO had varying effects on CXCL12 secretion (Supplementary Figure 10).

### AT101-induced suppression of CXCL12 is a function of its BH3 mimetic property

**Figure 2:**
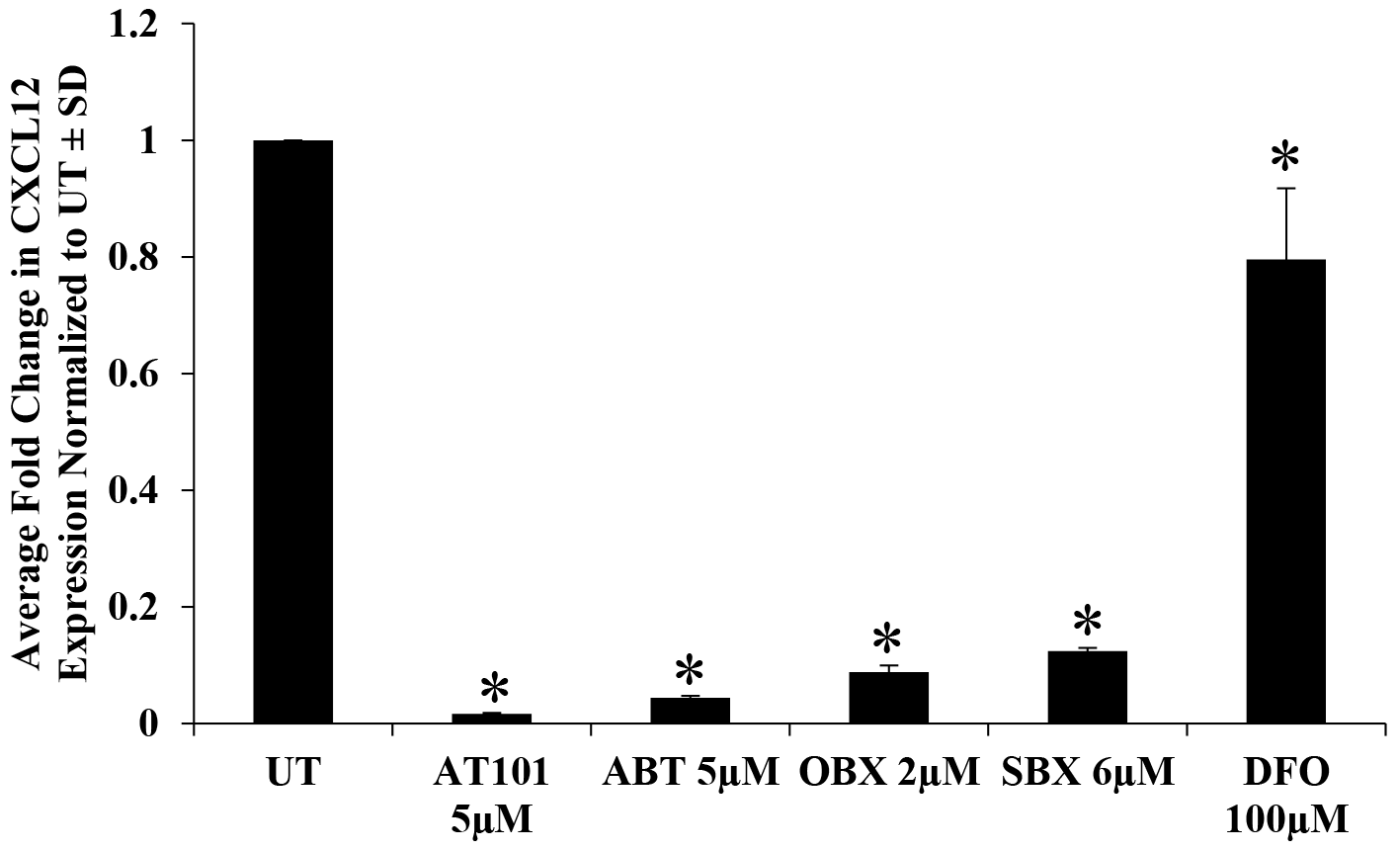
BH3 mimetics recapitulate the effects of AT101 on CXCL12 expression T265-2c cells treated with AT101, ABT, OBX, SBX or DFO significantly suppress CXCL12 mRNA levels compared to no treatment as demonstrated by qRT-PCR. *p-value <0.05. Comparison of DFO with AT101, ABT, OBX or SBX treatment resulted in a statistically significant difference in CXCL12 expression (p-value <0.01).

**Figure 3:**
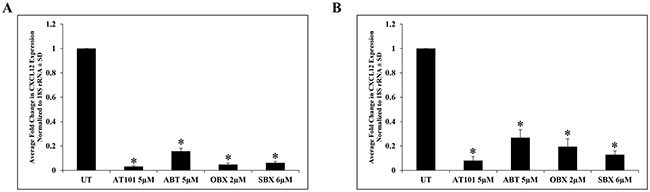
BH3 mimetic suppression of CXCL12 is conserved among multiple MPNST cell lines BH3 mimetics suppress CXCL12 mRNA levels in an NF1-derived (90-8) **A.** and sporadic (STS26T) **B.** MPNST cell line as demonstrated by qRT-PCR. *p-value <0.05.

**Figure 4:**
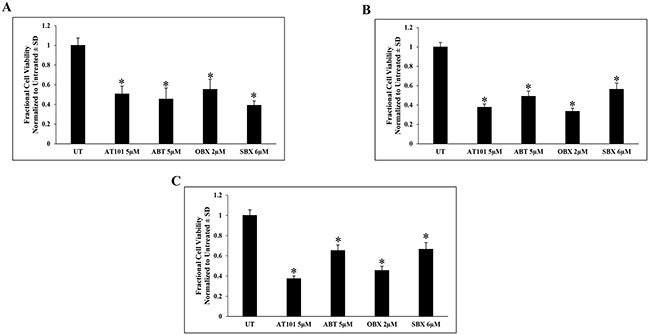
AT101 and BH3 mimetics mediate reduction in MPNST cell viability AT101- or BH3 mimetic-treated T265 **A.** 90-8 B. and STS26 C. MPNST cells demonstrate a decrease in viable cell number after 24h. *p-value <0.01.

Because AT101 has both BH3 mimetic and hypoxia mimetic effects [4], we sought to address which mechanism, if either, was responsible for the observed suppression of CXCL12 expression. We compared the effects of three BH3 mimetics (ABT, OBX, SBX) and a hypoxia mimetic (DFO) with AT101 on CXCL12 mRNA levels in T265-2c cells. BH3 mimetic drug concentrations were chosen because of the comparable reduction in viable cell number after 24h treatment. We found that all BH3 mimetics tested dramatically reduced CXCL12 mRNA levels after 24h (Figure 2, Supplementary Figure 6). DFO produced only a slight, albeit statistically significant, reduction in CXCL12 mRNA that was substantially less than that of BH3 mimetics (Figure 2, Supplementary Figure 6). These results suggest that BH3 mimetics as a class suppress CXCL12 expression and that AT101-mediated suppression of CXCL12 is not dependent on its ability to chelate iron. Further, to determine if CXCL12 suppression was a unique effect of BH3 mimetics on T265-2c cells or represented a more general response of MPNST cells, an additional NF1-derived (90-8) and a sporadic MPNST cell line (STS26T) were treated with AT101, ABT, OBX and SBX for 24h followed by qRT-PCR analysis of CXCL12. Both the NF1-derived (Figure 3A, Supplementary Figure 7) and sporadic (Figure 3B, Supplementary Figure 8) MPNST cell lines exhibited suppression of CXCL12 similar to T265-2c cells. These results suggest that BH3 mimetics possess a conserved function of CXCL12 suppression in MPNST cells. It is important to note that the BH3 mimetics tested exhibited conserved effects in U251 established human glioblastoma cells (Supplementary Figure 9). Further, BH3 mimetics reduced cell viability in all MPNST cell lines tested (Figure 4, Supplementary Figure 1/2/3) while DFO resulted in a less robust and reproducible effect (Supplementary Figure 11/12/13) Because CXCL12 is known to stimulate autocrine cell cycle progression via induction of cyclin D1, we evaluated cyclin D1 protein levels following AT101 or ABT treatment and observed an AT101- but not ABT-dependent reduction in cyclin D1 (Supplementary Figure 14).

### BH3 mimetics and PARP1

**Figure 5:**
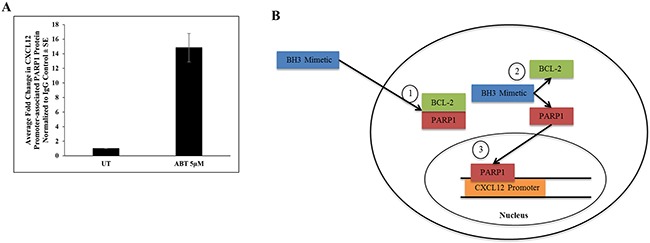
ABT increases PARP1 binding to the CXCL12 promoter **A.** T265-2c cells treated with ABT for 24h exhibit increased PARP1 binding to the CXCL12 promoter as demonstrated by qRT-PCR of Chromatin Immunoprecipitation (ChIP) with PARP1 antibody or IgG control. **B.** Proposed mechanism: PARP1 dimerizes with BCL-2 under normal physiological conditions (Step 1) (ref 33, ref 55). BH3 mimetics disrupt the BCL-2:PARP1 complex via the BH3 domain (Step 2) (ref 33), freeing PARP1 to bind the CXCL12 proximal promoter and exert transcriptional repression (Step 3).

The single most defining characteristic of Type 1 diabetes (T1D) is loss of pancreatic insulin-producing beta cells [28]. Because CXCL12 has demonstrated anti-diabetogenic capability by promoting pancreatic beta cell survival [31, 32], interest in the regulatory mechanism(s) of CXCL12 transcription has increased. A recent investigation into the transcriptional regulation of CXCL12 in rat pancreatic beta cells identified PARP1 as a negative transcriptional regulator of CXCL12 [28]. Interestingly, ABT has been shown to displace PARP1 from its interaction with the BCL-2 family member BCL-2 [33]. Considered together with the observation that PARP1 is known to have a negative regulatory effect on CXCL12 transcription [28], we hypothesized that at least some BH3 mimetic-mediated suppression of CXCL12 expression may result from liberation of a negative CXCL12 transcriptional regulator (PARP1). To test this hypothesis, we developed PCR primers flanking a putative PARP1 binding site within the CXCL12 promoter (Supplementary Figure 15) and performed ChIP analysis of T265-2c cells using antibodies against PARP1 or rabbit IgG following treatment with ABT. qRT-PCR analysis of ChIP products revealed a robust increase in PARP1 interaction with the *CXCL12* promoter (Figure 5A).

## DISCUSSION

The BCL-2 family of proteins mediates cellular life and death in part by regulating mitochondrial membrane integrity [34]. This regulation results from a delicate balance of interactions between BH domains of pro- and anti-apoptotic proteins within the BCL-2 family. Following various pro-death stimuli including DNA damage [35], growth factor deprivation [36] and oncogene activation [37], the balance of physical interactions between BH domains of pro- and anti-apoptotic proteins is disrupted by BH3-only proteins. This results in activation of pro-apoptotic multi-domain BCL-2 proteins (BAX/BAK) and subsequent apoptosis. It is estimated that roughly 50% of all human cancers exhibit elevated gene expression of anti-apoptotic BCL-2 family members [38], contributing to resistance to standard apoptosis-inducing therapies. Consequently, molecules possessing only the BH3 domain of BCL-2 proteins (termed BH3 mimetics), have received significant attention as potentially useful cancer therapeutic agents either alone or in combination with other drugs [34].

We have been investigating AT101, a well-known BH3 mimetic, as an alternative chemotherapeutic option for patients with MPNSTs. Previously published studies from our lab have demonstrated that AT101 can also chelate intracellular iron and induce a hypoxia-like state *in vitro*. qRT-PCR analysis demonstrated that AT101-induced suppression of CXCL12 transcription in all three MPNST cell lines tested. To gain insight into the mechanism by which AT101 exerts its suppressive effects on CXCL12, we compared AT101 with three BH3 mimetics (ABT, OBX, SBX) and a hypoxia mimetic (DFO). We found that BH3 mimetics, but not DFO, robustly suppressed CXCL12 in all three MPNST cell lines tested. These results suggest that AT101's ability to suppress CXCL12 is largely a function of its BH3 mimetic property rather than its iron chelation ability.

Understanding the mechanism behind BH3 mimetic-induced reduction of CXCL12 is critical to appropriate clinical application. To this end, we have investigated an interesting hypothesis based on the observation that ABT has been shown to displace PARP1 from BCL-2 [33] and that PARP1 exerts a strong inhibitory effect on CXCL12 transcription [28]. Our ChIP data, which demonstrates a robust BH3 mimetic-mediated increase in PARP1 binding to the *CXCL12* promoter, supports the hypothesis that disruption of the BCL-2:PARP1 interaction via BH3 mimetics results in increased negative regulation of CXCL12 transcription by PARP1. These data suggest that BH3 mimetic-mediated suppression of CXCL12 expression is, at least in part, mediated by displacement of PARP1 from its interaction with BCL-2 and subsequent binding of PARP1 to the *CXCL12* promoter resulting in transcriptional down-regulation (Figure 5B, proposed model).

Targeting tumor-specific growth promoting pathways is important for successful cancer therapy, and the CXCL12/CXCR4 signaling axis is involved in tumor progression and migration in multiple tumor types [12, 39, 40]. The physiological role for chemotactic cytokines is to direct the homing of hematopoietic cells to specific sites within the body via interaction with cell-surface receptors [41–47]. This physiological role may help explain the observation that organs possessing the highest frequency of breast cancer metastasis also have the most abundant secretion of these chemotactic ligands, suggesting a significant role for these molecules in breast cancer metastasis [11]. Supporting this notion is the observation that CXCL12 expression is elevated in multiple tissues known to harbor breast cancer metastases (lymph nodes, lung, liver, bone marrow) and relatively lower in tissues not typically associated with such metastases (small intestine, kidney, skin, brain, skeletal muscle) [11]. Further, *in vivo* neutralization of the CXCL12/CXCR4 interaction in breast cancer has been shown to significantly inhibit lung and lymph node metastasis [11]. Outside the context of cancer, reduction of CXCL12 has potential significance in the field of autoimmune disorders, as neutralization of chemokines is thought to have a restraining effect on the autoimmune response [48]. Conversely, disruption of the CXCL12/CXCR4 interaction has been shown to promote hematopoietic stem cell mobilization, promoting immunologic response of patients with non-Hodgkin lymphoma and multiple myeloma [49]. Additional studies are required to determine if BH3 mimetic suppression of CXCL12 is limited to MPNST and related tumor cells or is a general phenomenon with implications for other tumor types.

Affecting the CXCL12 signaling axis has potential for significant therapeutic benefit, as CXCR4 is over-expressed in more than 20 human cancers [50]. Accordingly, CXCR4 has been targeted by numerous antagonists including AMD3100, TN14003, T22, CTCE-9908 and ALX40-4C, all of which are CXCL12 N-terminus peptide analogs [51]. The ligand-dependent nature of this signaling axis relative to other cancer-relevant pathways of which ligand-independent signal transduction occurs via means such as receptor dimerization provides additional therapeutic targeting at the ligand level. This approach has potential for therapeutic benefit alone or as a combination approach to shut down the pathway at multiple levels. Here we demonstrate that BH3 mimetics as a class have the capability to robustly suppress CXCL12 expression and secretion in MPNSTs and set a precedent for further studies into the clinical utility of targeting the CXCL12 signaling axis by ligand ablation.

## MATERIALS AND METHODS

### Antibodies and other reagents

PARP1 primary antibody (#sc-7150 [H-250]) and rabbit IgG (sc-2027) were obtained from Santa Cruz (Dallas, TX). Other reagents were obtained from the following sources: ABT-737 [ABT] and Obatoclax [OBX] (Selleck Chemicals, Houston, TX S1002 and S1057, respectively), desferrioxamine [DFO] (Sigma D9533), sabutoclax [SBX] (AdooQ Biosciences, Irvine, CA A12823), AT101 (Ascenta Therapeutics, Malvern, PA).

### Cell culture

We have previously described the source of human NF1-derived MPNST cell lines (T265-2c, 90-8) utilized in this study [29]. STS26T sporadic MPNST cells were a gift from Dr. Steven Carroll whose source is described by Dahlberg *et al*. [30]. All cell lines were cultured in DMEM containing 1% penicillin/streptomycin (Invitrogen, Carlsbad, CA), 1% L-glutamine (Sigma), and 10% fetal bovine serum (Fisher Scientific). All cells were incubated at 37°C in a humidified 5% CO2, 95% air atmosphere. Based on cellular size and in order to achieve relative confluence, cell lines were plated at varying densities. For DNA, RNA and protein collection, cells were plated on 100mm dishes at a density of: 1×10^6^/dish- 90-8, STS26T; 1.5×10^6^/dish- T265-2c. Cultures were used in experiments 24 hours post-plating. Drug treatments were performed in respective media supplemented with 2% FBS.

### Cell viability assay

The calcein-AM conversion assay (Life Technologies, Carlsbad, CA C3100MP) was employed to quantify viable cell number after drug treatment as previously described [54, 55].

### ELISA

T265-2c cells were plated in 100mm dishes at previously described densities and were treated 24h post plating in media containing 2% FBS with or without AT101. Conditioned media was collected 24 hours after treatment and concentrated ~80-fold using Amicon Ultra-4 centrifugal filters with a molecular size exclusion of 3kD (Millipore, Billerica, MA UFC800324) and stored at -80°C. Following protein quantification, 375μg total protein was loaded per well (in duplicate) using a human CXCL12/SDF-1 ELISA kit (R&D Systems, Minneapolis, MN DSA00) per the manufacturer's instructions.

### Reverse transcription and real-time quantitative PCR analysis

RNA was isolated using the RNeasy Plus mini kit (Qiagen, Germany #74134). cDNA was subsequently synthesized using the High Capacity cDNA Reverse Transcription kit (Applied Biosystems, Foster City, CA #4368814). Real-time quantitative PCR was performed using the Maxima SYBR Green/ROX qPCR master mix (Thermo Scientific, Waltham, MA #K0221) and probes were obtained from Life Technologies (Carlsbad, CA). Amplifications were run in a StepOne Plus Real-Time PCR System (Applied Biosystems). The following sense/antisense primers and probes were selected from the Harvard PrimerBank and used for detecting: human CXCL12 (5’-ATTCTCAACACTCCAAACTGTGC-3’ and 5’-ACTTTAGCTTCGGGTCAATGC-3’) and human 18S rRNA (5’-GTAACCCGTTGAACCCCATT-3’ and 5’-CCATCCAATCGGTAGTAGCG-3’). Analysis was performed using the ΔΔCT method [52] and values were adjusted using 18S rRNA levels as reference.

### Chromatin immunoprecipitation (ChIP)

T265-2c cells were plated in 100mm dishes at densities described above in the *Cell Culture* section and drug treatments were performed after 24h. To account for loss of total cell number resulting from cytotoxic effects of BH3 mimetic treatment, twice as many 100mm dishes were used for each treatment condition versus untreated and each dish of respective cells was combined after fixation. ChIP using antibodies against PARP1 (Santa Cruz H-250 [sc-7150]) (12μg/ml) or rabbit IgG (Santa Cruz sc-2027) (12μg/ml) was performed using the ChIP-IT Express Enzymatic kit from Active Motif (Carlsbad, CA #53009) per the manufacturer's instructions. DNA was amplified using primers flanking the putative PARP1 [“A/GNNA/TCAAA” [53] binding site within the *CXCL12* promoter. Negative control primers were obtained from Active Motif (#103708).

Primer sequence:

Forward: 5′-GAATCTCCCGTCCCACTCC-3′

Reverse: 5′-GCCGAGCCTCAGTTTCCT-3′

### Statistics

All data points represent the mean ± standard deviation (S.D.) except for ChIP qRT-PCR data, which represents mean ± standard error (S.E.). All experiments were repeated at least 3 times (except for T265-2c ChIP and STS26T qRT-PCR) and representative data is shown; all biological replicates are included in supplementary material. Each real-time quantitative PCR was performed in triplicate and the average and S.D. (or S.E. for ChIP qRT-PCR) was used for statistical analysis. Each ELISA was performed in duplicate and averages from three biological and technical replicates were used to obtain an average and S.D. for statistical analysis. ELISA data is presented as a compilation of three replicate experiments. Statistical significance was determined by ANOVA followed by Dunnett's post-hoc test. A *p*-value < 0.05 was considered significant.

## SUPPLEMENTARY MATERIALS FIGURES



## Abbreviations

(MPNST): Malignant Peripheral Nerve Sheath Tumor
(NF1): Neurofibromatosis Type 1
(ABT): ABT-737
(OBX): obatoclax
(SBX): sabutoclax
(DFO): desferrioxamine
(BCL-2): B-Cell Lymphoma 2
(BH3): BCL-2 Homology domain 3
(PARP1): Poly (ADP-Ribose) Polymerase 1
(PLC): Phospholipase C
(PI3K): PhosphoInositide 3-Kinase
(MAPK): Mitogen-Activated Protein Kinase
(JNK): c-Jun N-terminal Kinase
