# Supplementary material for: BH3 mimetics suppress CXCL12 expression in human malignant peripheral nerve sheath tumor cells
Source: Oncotarget. 2016 Dec 31;8(5):8670–8. doi: 10.18632/oncotarget.14398 (PMC5352431; doi:10.18632/oncotarget.14398)
Supplement: Supplementary file 1 [file oncotarget-08-8670-s001.pdf]

BH3 mimetics suppress CXCL12 expression in human malignant peripheral nerve sheath tumor cells

SUPPLEMENTARY FIGURES

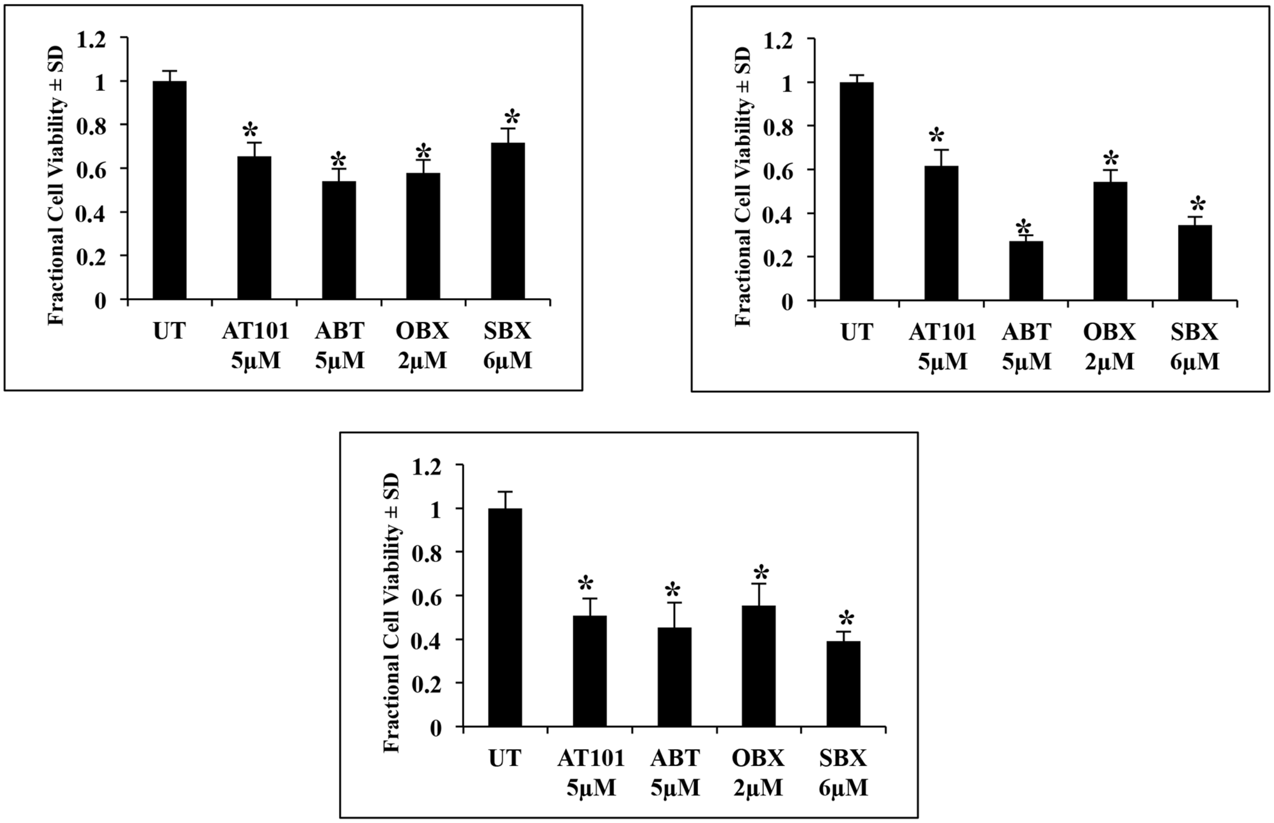

Supplementary Figure S1: AT101- or BH3 mimetic-treated T265-2c MPNST cells demonstrate a decrease in viable cell number after 24h as determined by calcein-AM conversion in three separate biological replicates. Data represented as mean +/- SD. *p<0.01.

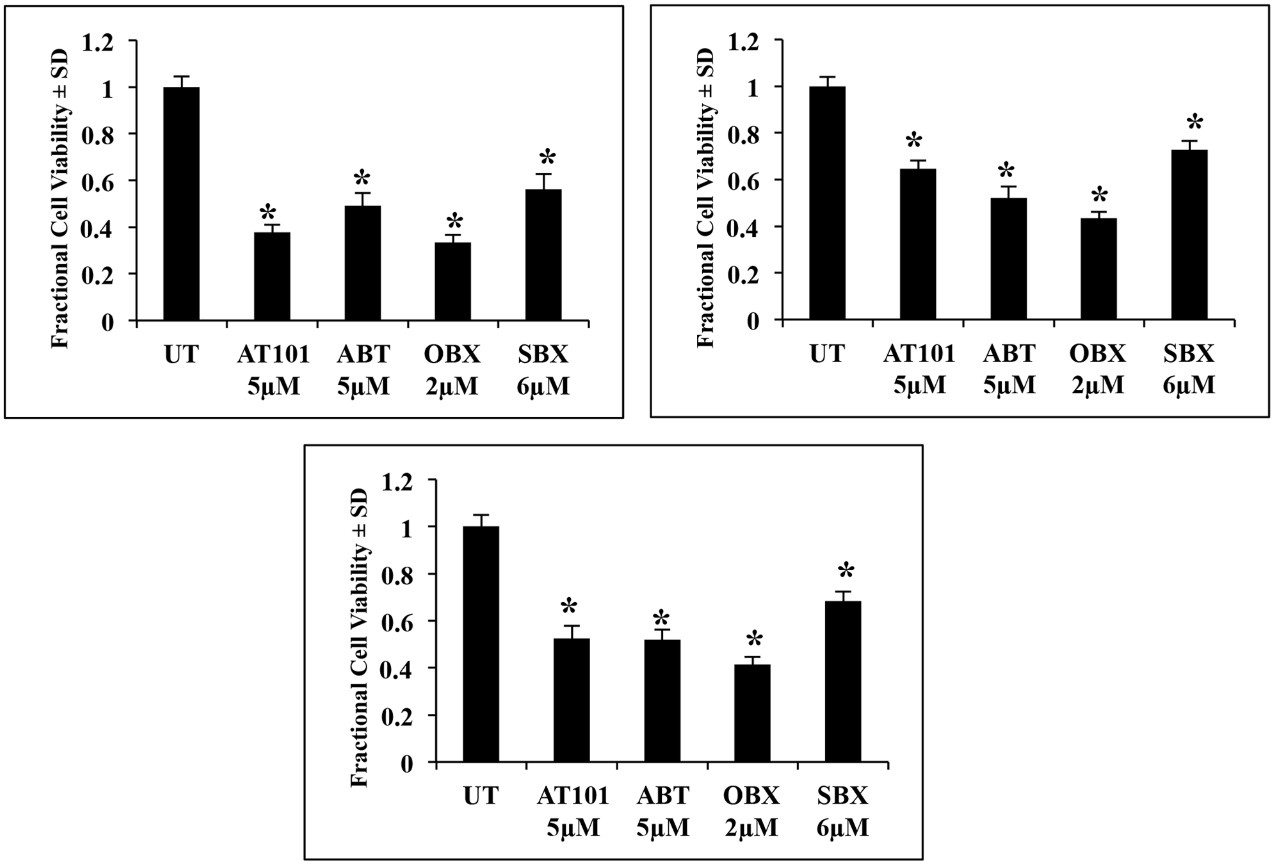

Supplementary Figure S2: AT101- or BH3 mimetic-treated 90-8 MPNST cells demonstrate a decrease in viable cell number after 24h as determined by calcein-AM conversion in three separate biological replicates. Data represented as mean +/- SD. *p<0.01.

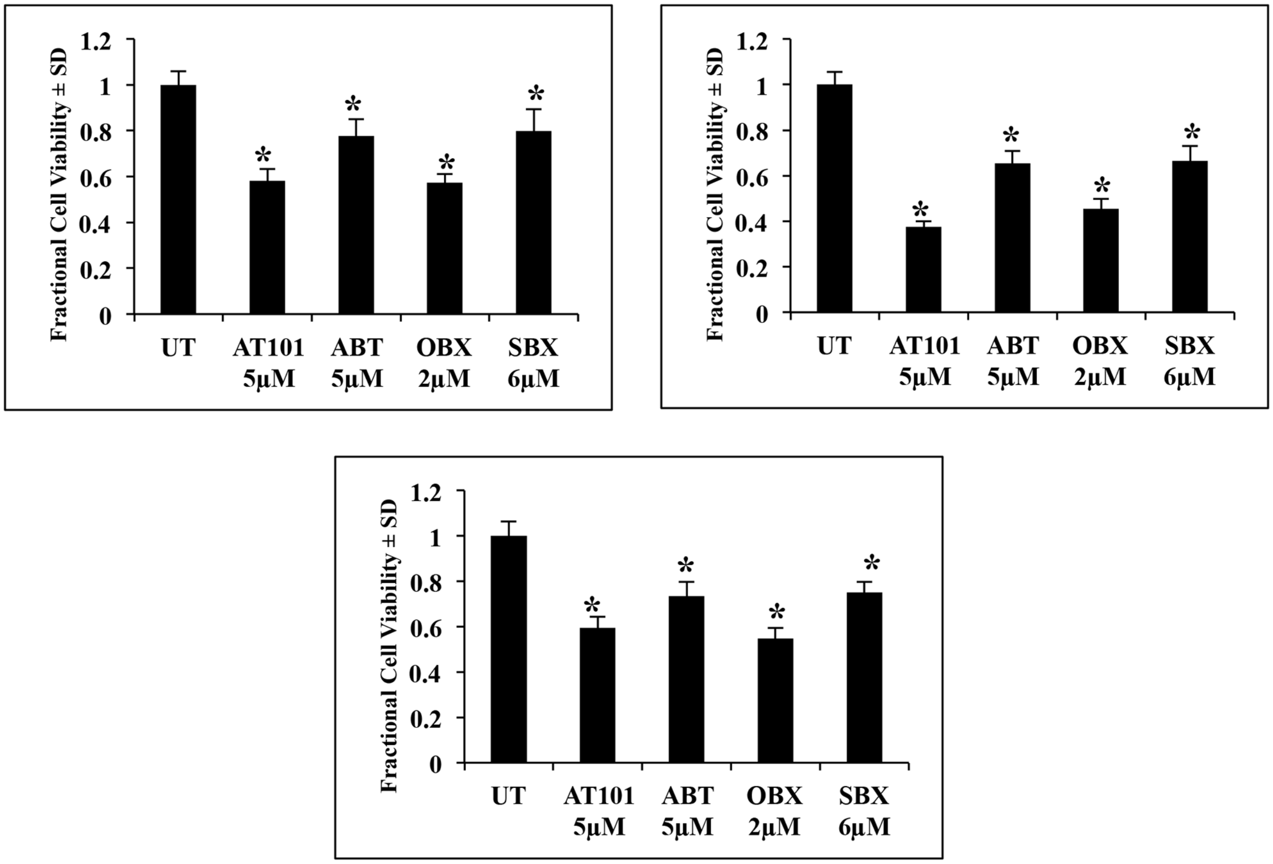

Supplementary Figure S3: AT101- or BH3 mimetic-treated STS26T MPNST cells demonstrate a decrease in viable cell number after 24h as determined by calcein-AM conversion in three separate biological replicates. Data represented as mean +/- SD. *p<0.01.

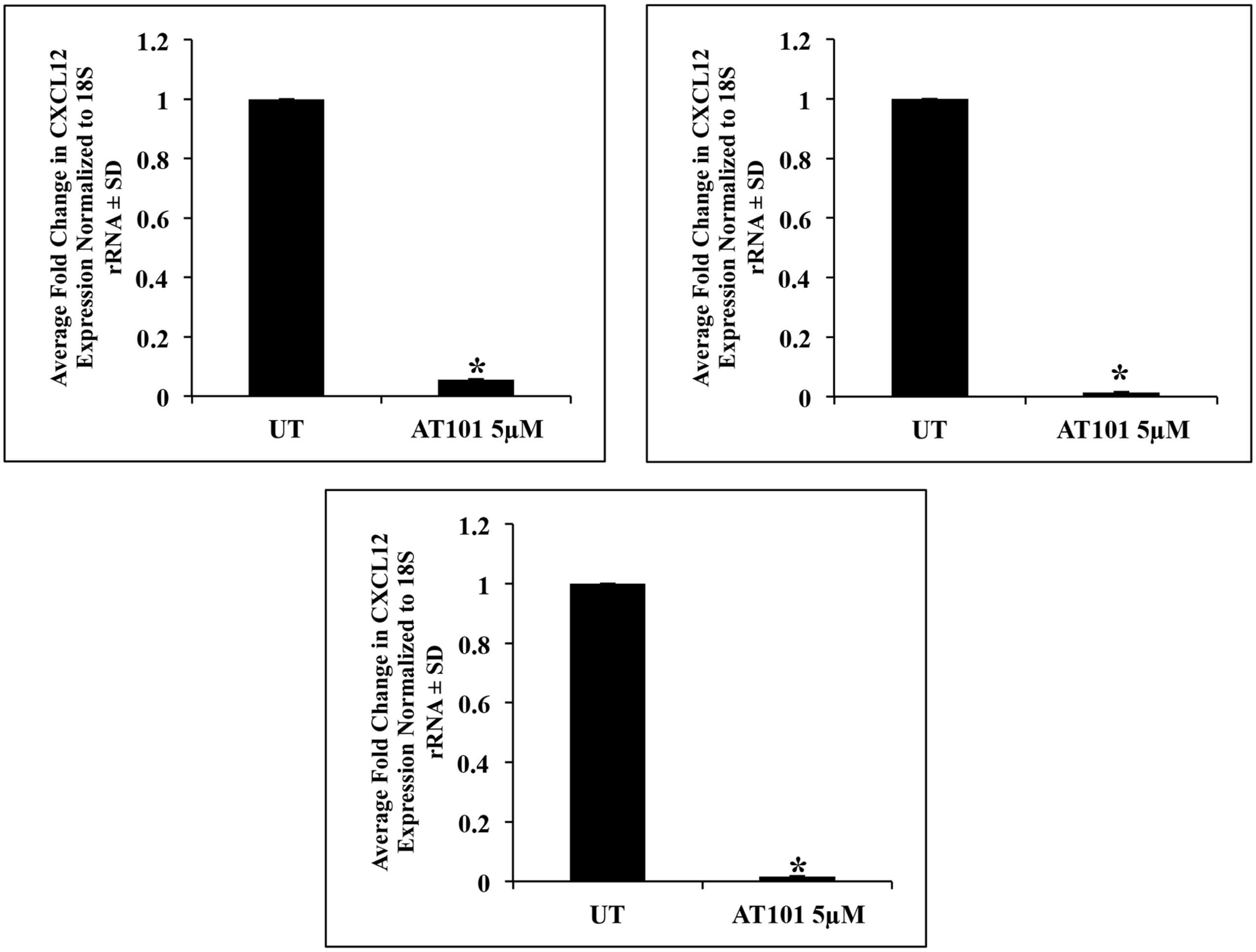

Supplementary Figure S4: AT101-treated T265-2c MPNST cells demonstrate a decrease in CXCL12 expression after 24h as determined by real-time quantitative PCR in three separate biological replicates. Data represented as mean +/- SD. *$p<0.01$.

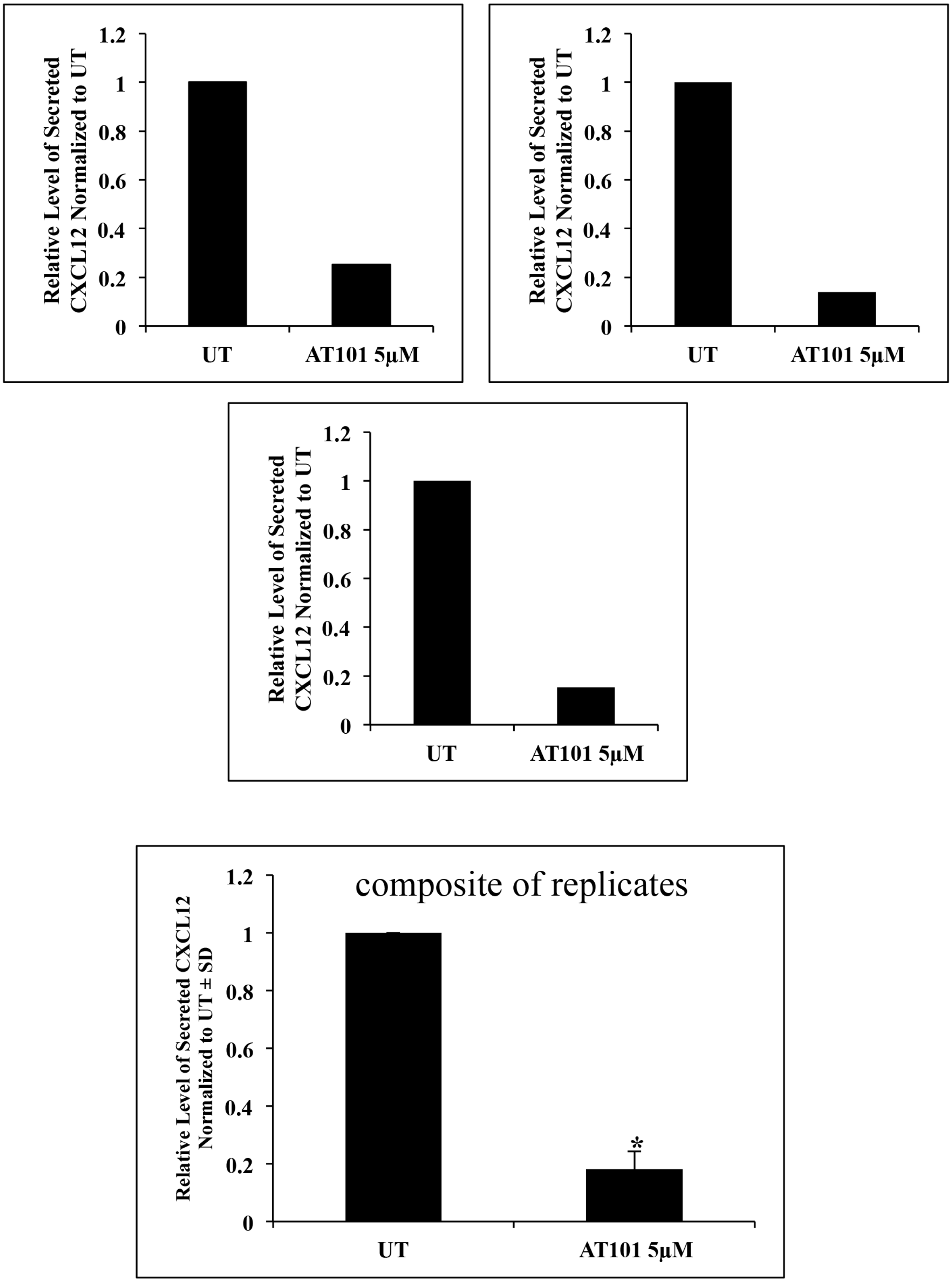

Supplementary Figure S5: AT101-treated T265-2c MPNST cells demonstrate a decrease in CXCL12 secretion after 24h as determined by CXCL12 Enzyme-Linked Immunosorbent Assay (ELISA) in three separate biological replicates. Data represented as mean +/- SD. *p<0.01.

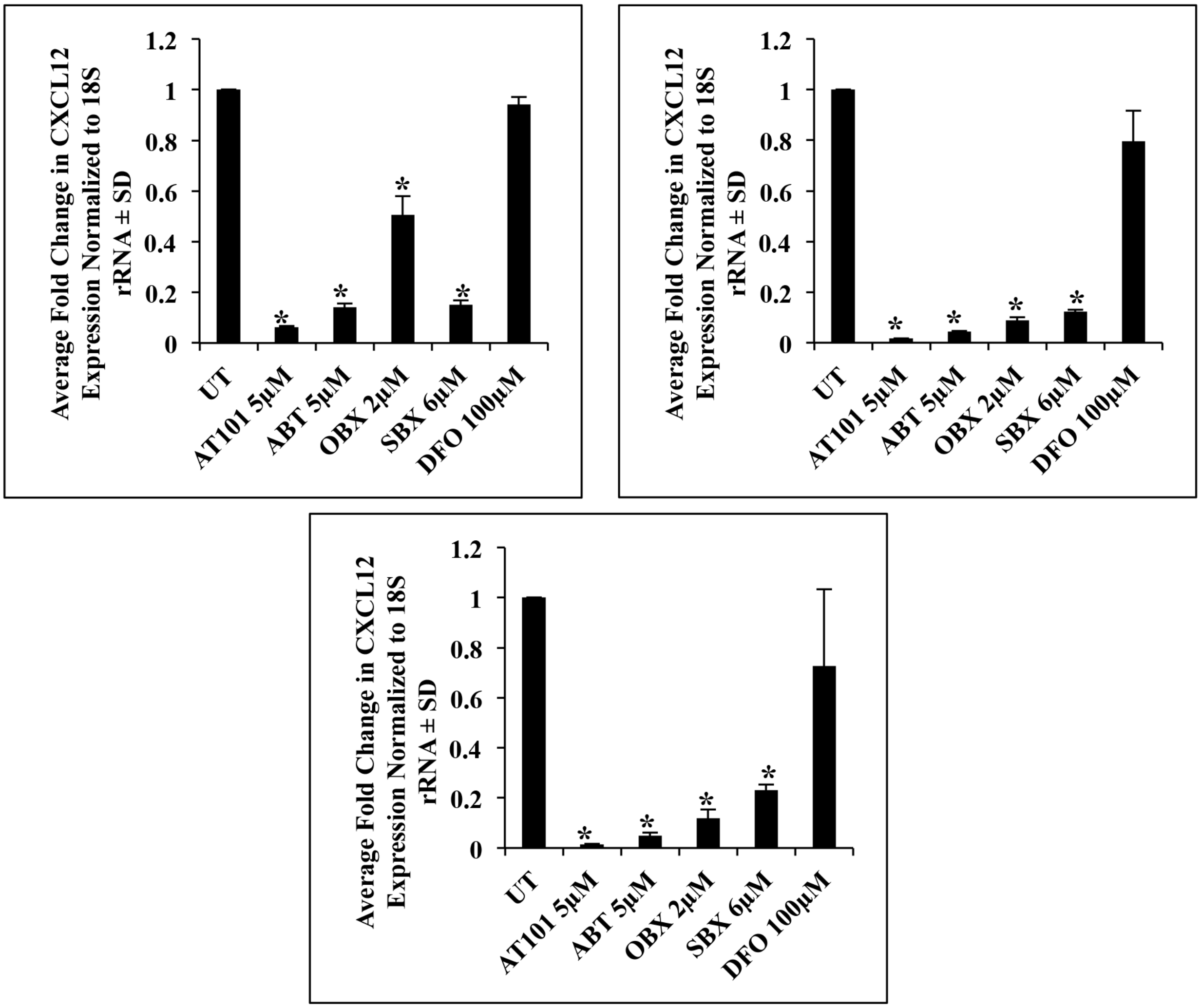

Supplementary Figure S6: AT101- or BH3 mimetic-treated T265-2c MPNST cells demonstrate a decrease in CXCL12 expression after 24h as determined by real-time quantitative PCR in three separate biological replicates. Data represented as mean +/- SD. *p<0.001.

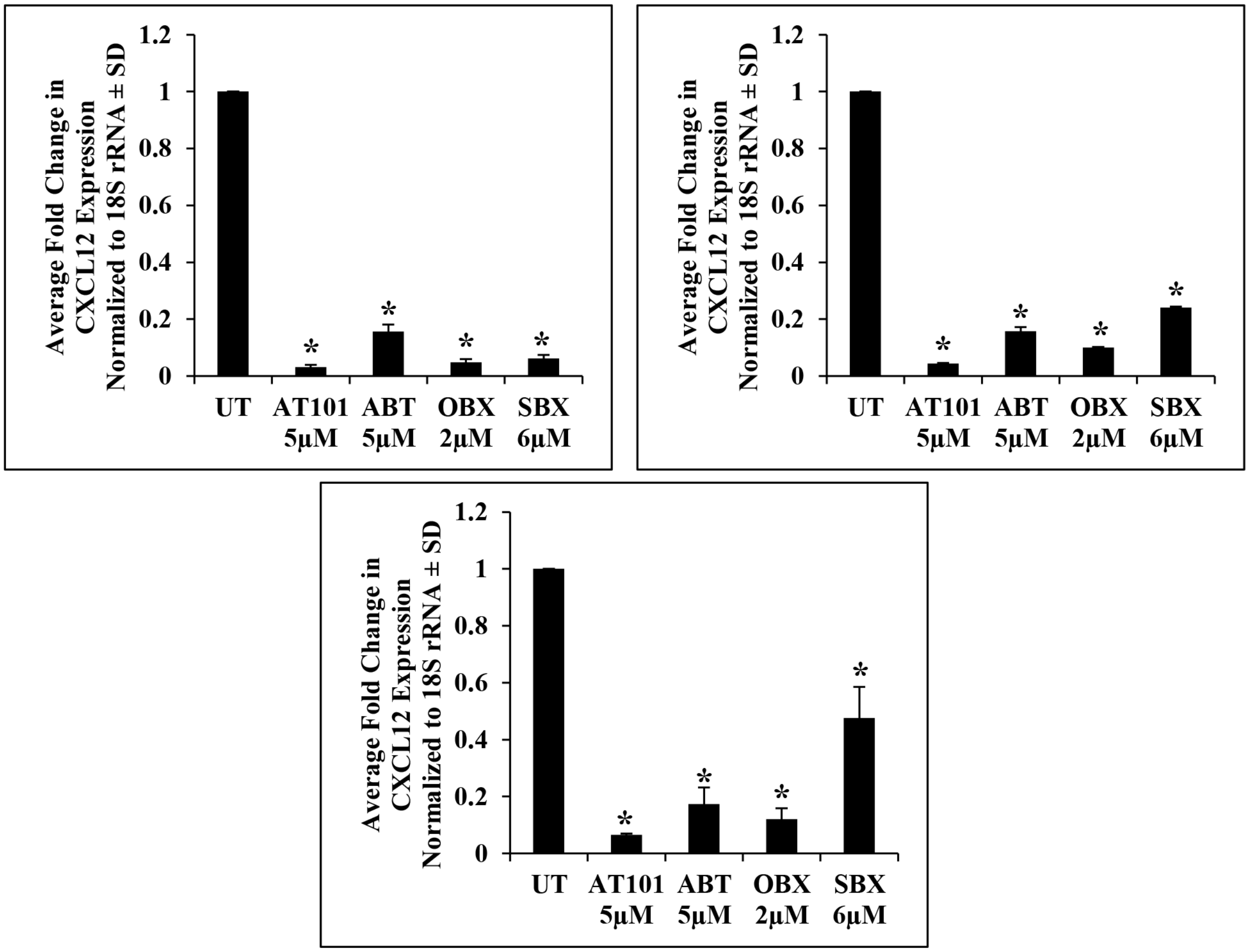

Supplementary Figure S7: AT101- or BH3 mimetic-treated 90-8 MPNST cells demonstrate a decrease in CXCL12 expression after 24h as determined by real-time quantitative PCR in three separate biological replicates. Data represented as mean +/- SD. *p<0.001.

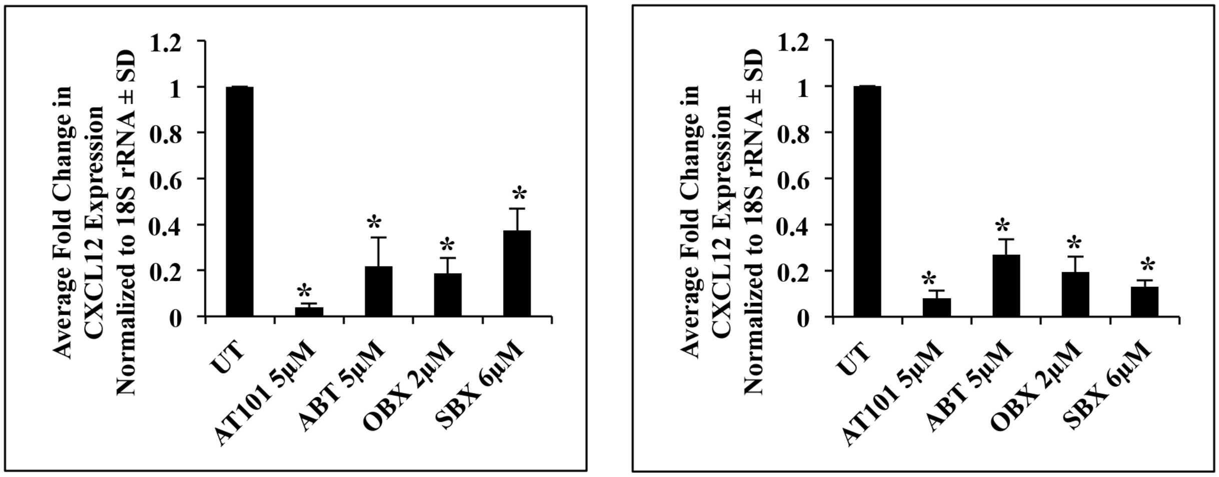

Supplementary Figure S8: AT101- or BH3 mimetic-treated STS26T MPNST cells demonstrate a decrease in CXCL12 expression after 24h as determined by real-time quantitative PCR in two separate biological replicates. Data represented as mean +/- SD. *p<0.001.

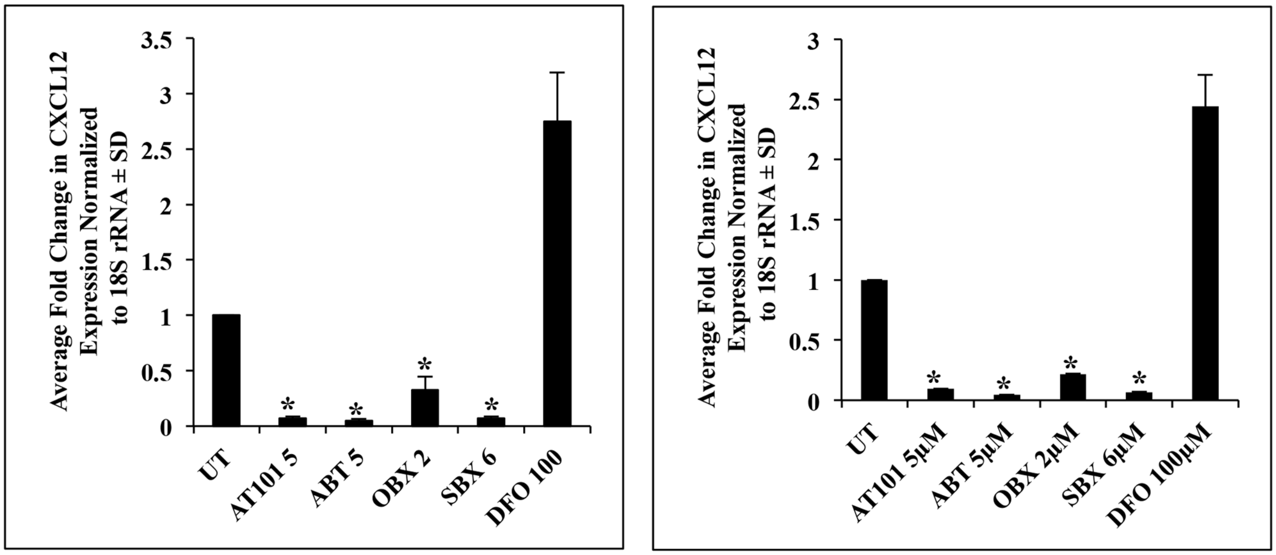

Supplementary Figure S9: AT101- or BH3 mimetic-treated U251 GBM cells demonstrate a decrease in CXCL12 expression after 24h as determined by real-time quantitative PCR in two separate biological replicates. Data represented as mean +/- SD. *p<0.001.

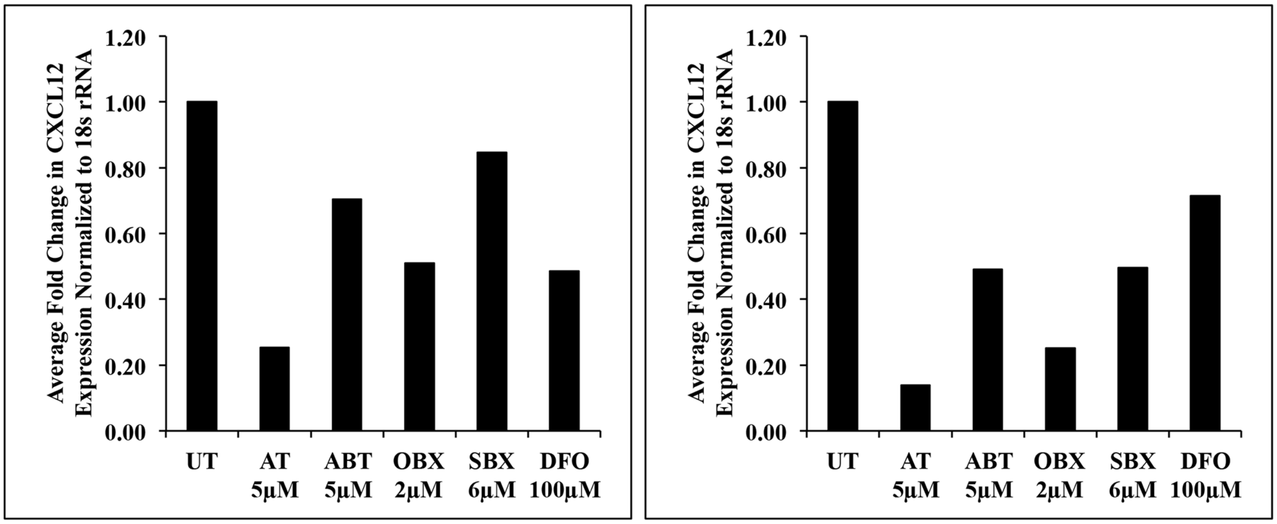

Supplementary Figure S10: AT101-treated T265 MPNST cells demonstrate a decrease in CXCL12 secretion after 24h while BH3 mimetic treatment has variable effects as determined by CXCL12 Enzyme-Linked Immunosorbent Assay (ELISA) in three separate biological replicates. No statistics were performed on these individual experiments.

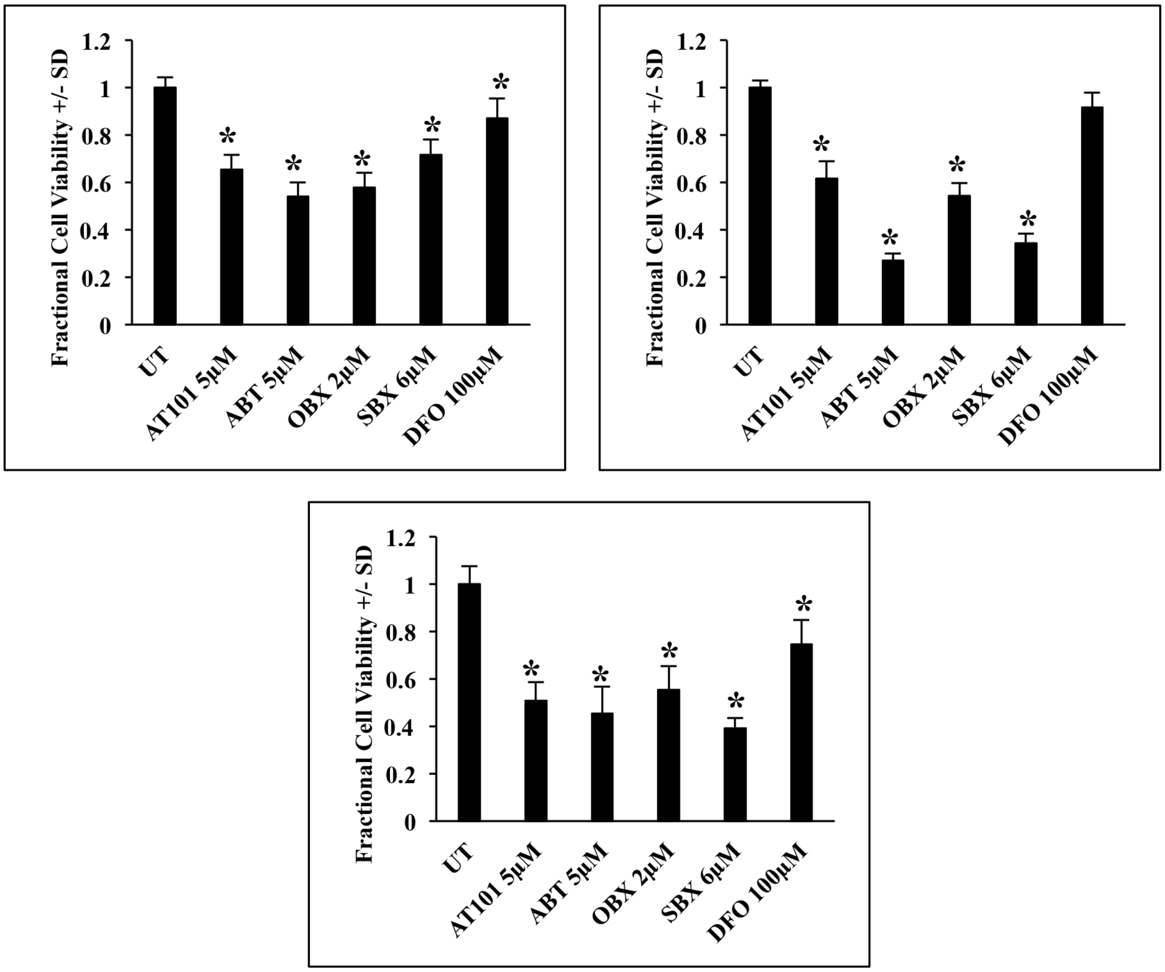

Supplementary Figure S11: AT101- and BH3 mimetic-treated T265 MPNST cells demonstrate a decrease in viable cell number while DFO treatment results in a less robust and reproducible effect after 24h as determined by calcein-AM conversion in three separate biological replicates. Data represented as mean +/- SD. *p<0.01.

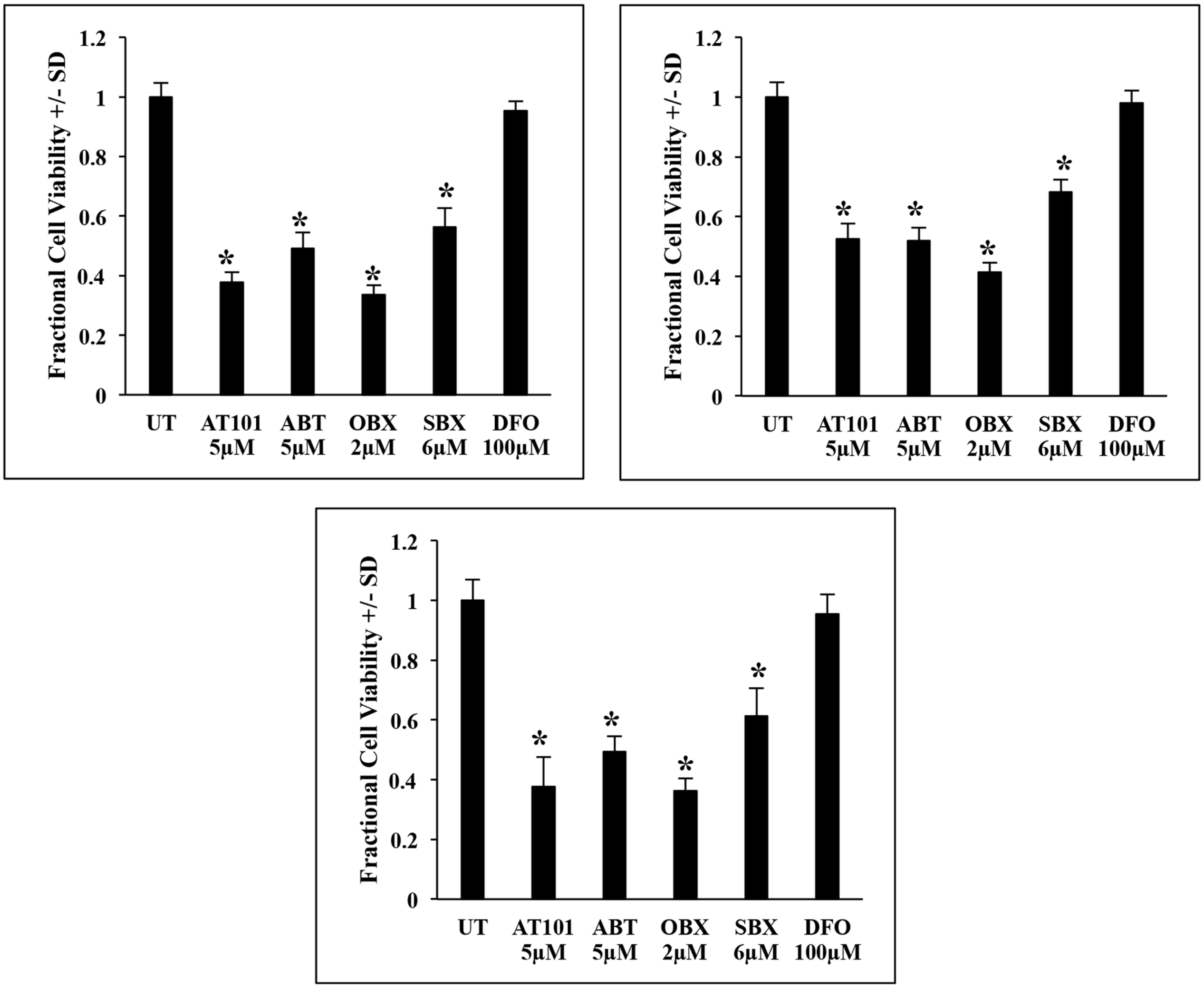

Supplementary Figure S12: AT101- and BH3 mimetic-treated, but not DFO-treated, 90-8 MPNST cells demonstrate a decrease in viable cell number after 24h as determined by calcein-AM conversion in three separate biological replicates. Data represented as mean +/- SD. *p<0.01.

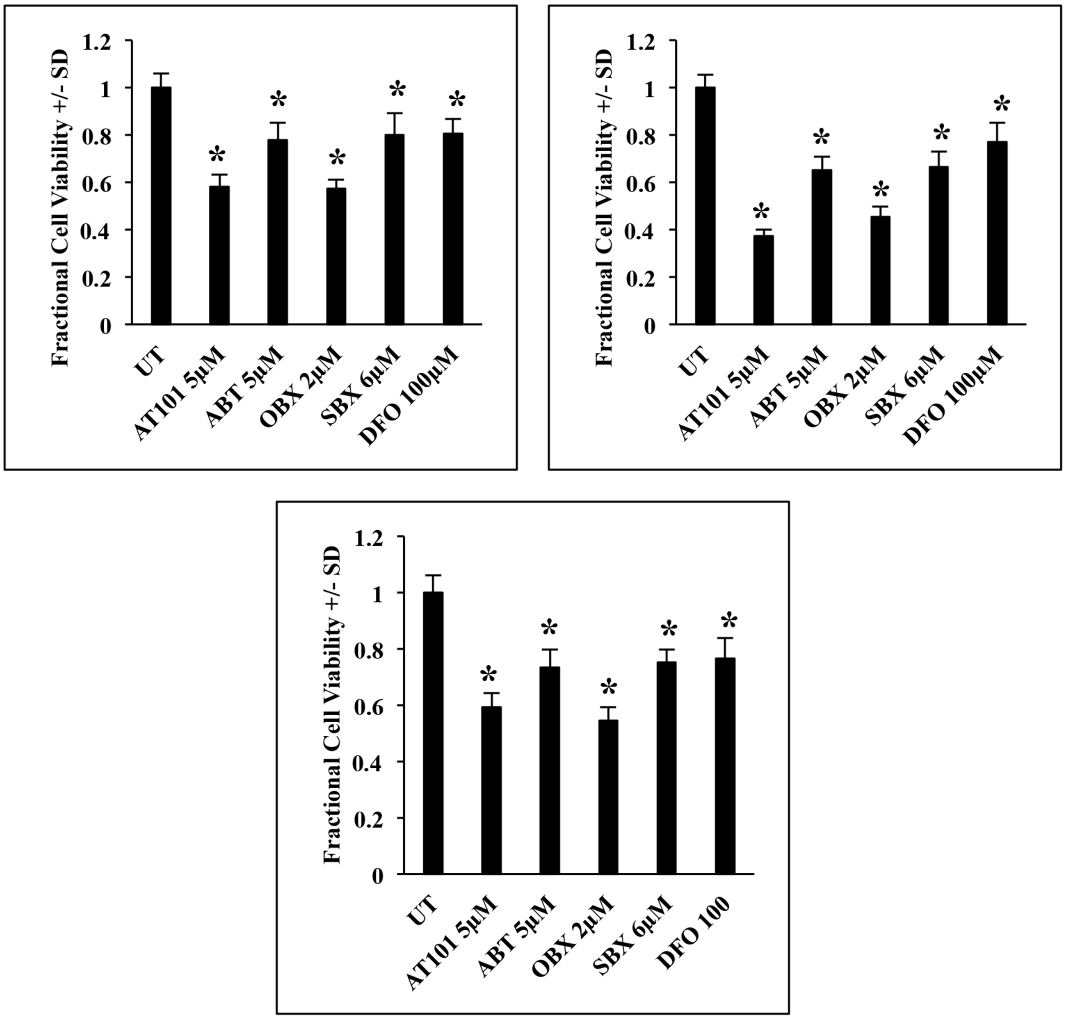

Supplementary Figure S13: AT101-, BH3 mimetic- and DFO-treated STS26T MPNST cells demonstrate a decrease in viable cell number after 24h as determined by calcein-AM conversion in three separate biological replicates. Data represented as mean +/- SD. *p<0.01.

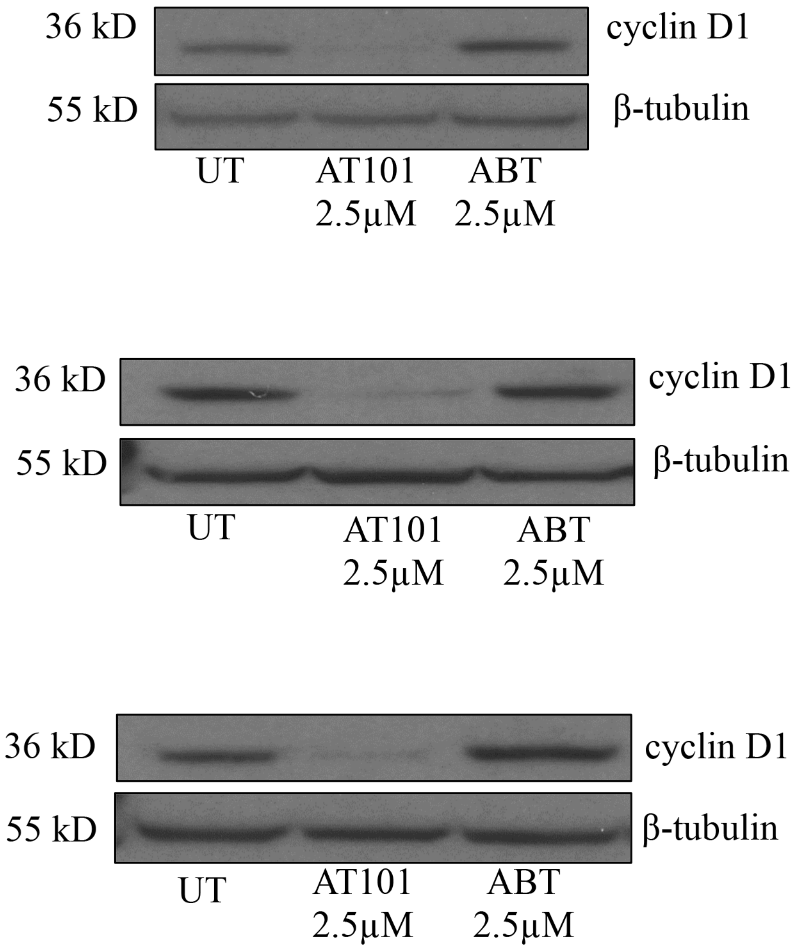

Supplementary Figure S14: T265 cells treated with AT101, but not ABT, exhibit a marked reduction in cyclin D1 protein after 24h as demonstrated by western blot in three separate biological replicates.

Primer Forward
-820 TGTCTGCTTGTCAGACACGATGCAGAGAATTTCGCGGCGTGGGGCGGGTCTCATTGAATCTCCCGTCCCACTCCGCGGGG

PARP1
-740 TGGGCCTGTGATTAGCTCATTTCACCATTGAGAGGTCGGAAGTACAAAGGCTACATTCGCTTTTACTGAGAGCCGCCGGCG

Primer Reverse
-660 CCTTCTGCTTTGTTTGTACAGGCGAGGAAACTGAGGCTCGGCTGGTGGCGCCGTGGGCTTGGAGTCCGAGCCACGCTGAC

-580 TGCAAAGACGGGTCTCATTCCCGCAGATCGAGCTCTGCCGGCGGCTGCGCCGCAAGCCGGGCAGGTGGCGAGCTTGAGC

-500 CCCCACGCACAGAAAGCAGGACCCCCTCGGCTGCCTTGGGCCGCCACCGCCAGCAGGCCCTCCGCCCGGGACTAACTTG

-420 TTTGCTTTTCATTGGTTCTCATTCAGTTCCCGCCATCGAAAGGCCCCGTCCCGCAGCTTTCCACGCGCGCCCCACTTTACGC

-340 CTAAGGTCCTCAGTCTCTCCAGTGGGGCCCTGTCACAGGGACAATAAGCGGCCCTCCAGCCGGCGTCGCTCAGGCTGCGG

-260 ACCTCACTGCAGACCGGGCCAGCGGTGCGGGGCCCAGCGGAGCCTGAGAAGGTCAAAGGCCGGAGCGCACTGCGCCTC

-180 GGGAGCACAGAGGGAGCGGAGGAGGGGCGAAGGGGATGGGTGGGGGGTGCCGCCGAGGGAGTCGCGCGTCAGAGACC

TATA
-100 CCGGCCACGGCCAGCACTCGGCTCCGGGCCCGCCCCTCACCGCGCGCCCCGCCCCGCCCCGCCTGGCTCTCCCCTCTAAA

+1
-20 GCGCCCGGCGCGCGCCTCCCACCGCCGCACTTTCACTCTCCGTCAGCCGCATTGCCCGCTCGGCGTCCGGCCCCCGACCC

ORF
+61 GCGCTCGTCCGCCCGCCCGCCCGCCCGCCCGCGCCATGA...3′

Supplementary Figure S15: PARP1 proximal promoter primer design for ChIP.
